# Anti-angiogenic effects of crenolanib are mediated by mitotic modulation independently of PDGFR expression

**DOI:** 10.1038/s41416-019-0498-2

**Published:** 2019-06-25

**Authors:** Robert H. Berndsen, Cédric Castrogiovanni, Andrea Weiss, Magdalena Rausch, Marchien G. Dallinga, Marijana Miljkovic-Licina, Ingeborg Klaassen, Patrick Meraldi, Judy R. van Beijnum, Patrycja Nowak-Sliwinska

**Affiliations:** 1 Molecular Pharmacology Group, School of Pharmaceutical Sciences, University of Lausanne and University of Geneva, Rue Michel-Servet, 1211 Geneva, Switzerland; 2 Angiogenesis Laboratory, Department of Medical Oncology, Cancer Center Amsterdam, Amsterdam UMC—location VUmc, VU University Amsterdam, De Boelelaan, 1117 Amsterdam, The Netherlands; 3 Department of Cell Physiology and Metabolism, University of Geneva Medical School, Geneva, Switzerland; 4 Ocular Angiogenesis Group, Departments of Ophthalmology and Medical Biology, Amsterdam Cardiovascular Sciences, Cancer Center Amsterdam, Amsterdam UMC, University of Amsterdam, Meibergdreef 9, Amsterdam, The Netherlands; 5 Department of Pathology and Immunology, University of Geneva Medical School, Geneva, Switzerland; 6 Translational Research Center in Oncohaematology, Rue Michel-Servet, 1211 Geneva, Switzerland

**Keywords:** Drug development, Cancer microenvironment

## Abstract

**Background:**

Crenolanib is a tyrosine kinase inhibitor targeting PDGFR-α, PDGFR-β and Fms related tyrosine kinase-3 (FLT3) that is currently evaluated in several clinical trials. Although platelet-derived growth factor receptor (PDGFR) signalling pathway is believed to play an important role in angiogenesis and maintenance of functional vasculature, we here demonstrate a direct angiostatic activity of crenolanib independently of PDGFR signalling.

**Methods:**

The activity of crenolanib on cell viability, migration, sprouting, apoptosis and mitosis was assessed in endothelial cells, tumour cells and fibroblasts. Alterations in cell morphology were determined by immunofluorescence experiments. Flow-cytometry analysis and mRNA expression profiles were used to investigate cell differentiation. In vivo efficacy was investigated in human ovarian carcinoma implanted on the chicken chorioallantoic membrane (CAM).

**Results:**

Crenolanib was found to inhibit endothelial cell viability, migration and sprout length, and induced apoptosis independently of PDGFR expression. Treated cells  showed altered actin arrangement and nuclear aberrations. Mitosis was affected at several levels including mitosis entry and centrosome clustering. Crenolanib suppressed human ovarian carcinoma tumour growth and angiogenesis in the CAM model.

**Conclusions:**

The PDGFR/FLT3 inhibitor crenolanib targets angiogenesis and inhibits tumour growth in vivo unrelated to PDGFR expression. Based on our findings, we suggest a broad mechanism of action of crenolanib.

## Background

Targeting angiogenesis currently is a well-established approach in cancer therapy. Key players in the process of angiogenesis include vascular endothelial growth factor receptor (VEGFR)-, fibroblast growth factor receptor (FGFR)- and TIE2 receptor signalling.^1^ The role of platelet-derived growth factor receptor (PDGFR) signalling in angiogenesis is not fully defined although it has been reported to contribute to angiogenesis and other mechanisms including cell growth, differentiation and migration.^2,3^ Studies on the effects of PDGFR signalling on angiogenesis in both in vivo and in vitro models, as well as its contribution to the recruitment of pericytes in tumours,^4^ suggest an important role in the development and maintenance of functional vasculature.^5^

In order to improve anti-angiogenic therapy, it is important to identify drugs that target angiogenesis. For instance, combination of specific drugs that target angiogenesis via non-parallel pathways may increase clinical efficacy.^6^ Furthermore, detailed knowledge of the mechanism of action and identification of cell types that are affected by specific drugs can help design more effective treatment approaches. Although most tyrosine kinase inhibitors (TKIs) have been developed to target either one or a small number of signalling pathways it is becoming more clear that such drugs have a significant amount of off-target interactions.^7^ This may result in unexpected efficacy in less obvious tumour types on the one hand and insight in mechanisms of drug toxicities on the other hand.

Crenolanib was designed as a specific and selective PDGFR inhibitor,^8^ though more recently it was shown to also target FLT3.^9^ It was first clinically tested in a phase I dose escalation study in patients with advanced solid tumours in 2009 and was considered safe and well tolerated.^8^ Currently, the efficacy of crenolanib is being evaluated in clinical trials for several indications including gliomas with PDGFR-α amplifications (NCT02626364), oesophageal cancers (NCT03193918), relapsed/refractory FLT3 mutated-positive acute myeloid leukaemia (AML; NCT02298166) and gastrointestinal stromal tumours (GIST; NCT02847429).

Given the proposed importance of PDGF(R) in vascular homoeostasis, in this study we set out to investigate the effects of crenolanib on endothelial cells (EC) in comparison to tumour cells and fibroblasts. We show that crenolanib strongly affects EC migration and sprout length, partly by differentially affecting endothelial tip cells. In addition, crenolanib affects mitosis and induces apoptosis. In silico approaches provided supporting evidence for alternative pathways that crenolanib may act on to induce these effects. We further show that crenolanib inhibits pericyte recruitment that hampers formation of capillary-like networks. In vivo, this resulted in tumour growth inhibition, accompanied by a significant reduction in microvessel density (MVD).

## Materials and methods

### Compounds

Crenolanib was purchased from Selleck Chemicals (Houston, Texas, USA) and was dissolved in sterile DMSO at a concentration of 10 mg/ml. Aliquots were stored in −80 °C and thawed prior to each experiment. The maximum DMSO percentage was 0.1% and showed negligible activity in the performed assays and was used as control (CTRL).

### Cells

Immortalised human vascular endothelial cells (ECRF24) were cultured in flasks coated with 0.2% gelatine and grown in medium containing 50% DMEM and 50% RPMI-1640 (Life Technologies, Carlsbad, CA, USA), supplemented with 10% fetal bovine serum (FBS) and 1% penicillin/streptomycin (Life Technologies). Human ovarian carcinoma cells (A2780) and adult human dermal fibroblasts (HDFa) were cultured in DMEM supplemented as described above. Human umbilical vein endothelial cells (HUVEC) were harvested from umbilical cords and cultured in RPMI-1640 medium supplemented with 10% human serum, 10% FBS, 1% penicillin/streptomycin and 2 mM L-glutamine.^10^

### Cell viability and endothelial cell sprouting assay

For cell viability experiments, cells were seeded in 96-well cell culture plates at a density of 5 × 10^3^ cells/well (HUVEC) or 10 × 10^3^ cells/well (ECRF24 and A2780) and grown for 24 h.^11^ After the administration of test compounds, cells were allowed to grow for 72 h. After that cell viability was assessed with the CellTiter-Glo luminescence assay (Promega, Madison, WI, USA). Cell response to drug treatment was determined based on normalising the luminescence signal in the treated wells as compared to controls.

For the 3D sprouting assay, HUVEC were mixed in methylcellulose containing medium (70% RPMI, 20% Methocel^TM^/RPMI-1640 (Sigma-Aldrich, St. Louis, MO, USA) and 10% human serum).^12^ Drops of 1000 cells in 25 µl were deposited on the lid of a petri dish, which was then flipped to allow spheroid formation in hanging drops, and incubated overnight. After 24 h, a collagen gel mixture was prepared using PureCol^®^ (Sigma), 0.2 M NaOH and M199 medium (Sigma), new-born calf serum (NBCS), heparin and bFGF (50 ng/ml;). Spheroids were collected by flushing the lid with PBS, spun (400 g for 5′), gently mixed with the collagen gel solution and placed in pre-warmed Ibidi (Martinsreid, Germany) culture slides. Images of spheroids were taken after overnight incubation using a Leica DMI3000 microscope (Leica, Rijswijk, Netherlands). Image-based quantification was performed using ImageJ software.^12^

### Cell migration assay

ECRF24 (30 × 10^3^ cells/well) or HUVEC (15 × 10^3^ cells/well) were seeded in 96-well cell culture plates and grown overnight to confluency. A uniform scratch was made using a sterile scratch tool (Peira Scientific Instruments, Beerse, Belgium) and treatment was administered immediately after. The wells were imaged using a Leica DMI3000 microscope (Leica) at ×5 magnification using Universal Grab 6.3 software (DCILabs, Keerbergen, Belgium). Imaging was performed immediately (*T* = 0) and 6 h (*T* = 6) after scratching. The size of the scratch was automatically quantified and analysed using Scratch Assay 6.2 (DCILabs) by calculating the absolute wound closure (initial minus final scratch surface) and values were presented as the percentage normalised to the CTRL (0.1% DMSO in cell culture medium).

### Endothelial–Pericyte co-culture network formation assay

Adherent HUVEC and primary pericytes were labelled with 1 μM CellTracker Orange CMRA548 (Thermo Fisher Scientific, Waltham, MA, USA) and 1 μM CellTracker Green CMFDA488 (Thermo Fisher Scientific) dyes, respectively, in serum-free M199 medium for 30 min at 37 °C. When premixed with growth factor-reduced Matrigel (Corning, New York, NY, USA) both HUVEC and pericytes were then harvested by trypsinisation and counted. 2.5 × 10^3^ HUVEC and 5 × 10^3^ pericytes were added to the polymerised Matrigel in each well and cultured in complete M199 for up to 10 h. The co-cultures were analysed by live-cell time-lapse imaging using Nikon A1R confocal microscope (see Supplementary Methods).

### Flow cytometry

Analysis of cellular DNA content using propidium iodide (PI) was performed using flow cytometry.^11^ Cells were seeded at 20–40 × 10^3^ cells/well and incubated for 24 h. Medium with or without or crenolanib was applied and cells were incubated for an additional 72 h. Cells were harvested by trypsinisation and fixated in 70% ethanol for 2 h at −20 °C. Cell pellets were then resuspended in DNA extraction buffer (90 parts 0.05 M Na_2_HPO_4_, 10 parts 0.025 M citric acid, 1 part 10% Triton-X100, pH 7.4) and incubated for 20 min at 37 °C. Propidium iodide (PI, 20 μg/ml) was added and cells were analysed with a FACSCalibur flow cytometer (BD Biosciences, Franklin Lakes, NJ, USA). DNA content was quantified with CellQuest Pro software (BD Biosciences).

### Mitosis live-cell imaging

To visualise the effect of crenolanib on mitosis, live-cell imaging was performed for 24 h at 37 °C on a Ti widefield microscope (Nikon) equipped with an environmental chamber (5% CO_2_) using a 60 × 1.3 NA oil objective, a Cy5 filter, a CoolSNAP HQ camera (Roper Scientific, Vianen, Netherlands) at a sampling rate of 3 min, recording at each time point 9 z-stacks separated by 2 µm. ECRF24 were maintained as described above. HUVECs were maintained in M199 medium (Thermo Fisher Scientific) supplemented with 10% fetal calf serum (FCS), 1% penicillin/streptomycin (Thermo Fisher Scientific), 1% endothelial cell growth supplement (Millipore), 0.1 mg/mL heparin sodium salt (Sigma), 0.1 µM hydrocortisone (Sigma), and 10 µg/mL L-ascorbic acid (Sigma). The day before, the cells were seeded in an Ibidi µ-Slide 8 Well culture plate (Vitaris, Baar, Switzerland). 4 h prior to the start of imaging, 50 nM SiR-Tubulin (SpiroChrome AG, Switzerland) was added on the cells to stain microtubules in addition with 10 µM Verapamil (SpiroChrome AG, Stein am Rein, Switzerland) to keep the dye inside the cells. None of these compounds affected mitosis, nor the efficacy of crenolanib. Crenolanib was added just before the start of image acquisition. Time-lapse movies were analysed using NIS Elements AR Software.

### Immunofluorescence

In ECRF24, HUVEC, A2780 and HDFa F-actin and cell nuclei were visualised by a combination of phalloidin-Alexa488 (A12379, Invitrogen) and DAPI (D9542, Sigma). Separately, HUVEC were stained for VE-cadherin and DAPI (detailed in Supplementary Material).

### Human ovarian carcinoma grown and photodynamic therapy on the chicken chorioallantoic membrane

Fertilised chicken eggs were incubated in a hatching incubator (relative humidity 65%, 37^o^C). On embryo development day (EDD) 8, 25 μL hanging drops containing 10^6^ A2780 cells in 20% Methocel^TM^ (Sigma) and 80% serum free RPMI-1640 medium were prepared. Three hours later the spheroids were transplanted onto the surface of the chicken chorioallantoic membrane (CAM).^11,13^ Vascularised three-dimensional tumours were visible and eggs were randomised on EDD 11. Crenolanib, freshly dissolved in 0.9% NaCl, was administered on EDD 11 and 12 (referred to as treatment day 1 and 2) in 100 µl i.v. injections. The injected doses (52 μg/kg/day and 260 μg/kg/day) were adjusted to the normalised embryo weight at EDD 11 and 12. Control tumours were treated with vehicle (0.1% DMSO in 0.9% NaCl). Tumours were monitored daily for 8 days and tumour size was calculated with the formula: volume = [large diameter] × [perpendicular diameter]^2^ × 0.52. At the last experiment day, embryos were sacrificed and weighed. Tumours were resected and fixed in zinc-fixative for additional analysis. In vivo angiogenic sprouting on the CAM was induced by photodynamic therapy (PDT) and visualised on EDD 11.^14^ Directly after PDT, 20 μl crenolanib at a dose of 25 μM (corresponding to 63.5 μg/kg) was administered intravenously. Fluorescence images were taken 24 h later using a pco.1300 12-bit CCD camera (Gloor Instruments AG, Usler, Switzerland) run by Micro-Manager 1.4 (NIH, Bethesda, MD, USA).^11,15^

### Immunohistochemistry

Immunohistochemical staining of A2780 CAM tumours was performed to detect blood vessels (CD31) and proliferating cells (Ki67; Supplementary Methods). Microvessel density (MVD; number of CD31+ structures per microscopic field) and the frequency of Ki67 positive cells in CAM tumours was assessed by ImageJ quantification of representative images (×20 objective) using the colour deconvolution plugin, as previously described.^16^

### In silico analysis of crenolanib target proteins

Crenolanib target proteins were retrieved from proteomicsDB (www.proteomicsdb.org), an online repository on the human proteome. Using the ‘Analytics Toolbox’ function, dose-dependent protein-drug interactions can be mined. We used a concentration of 5 μM crenolanib to search for target proteins, and proteins reported with an effective inhibition of ≥50% with this concentration of crenolanib were subsequently included for further analysis. More details are provided in Supplementary Methods.

### Statistics and data correction

The data are presented as the mean of multiple independent experiments (±SEM). In the MVD analysis, statistical outliers were removed from the dataset using the modified thompson Tau test. Statistical significance was determined using the one-way or two-way ANOVA test with post hoc Dunnett’s multiple comparison test or an unpaired *t*-test (Graphpad Prism). *P values lower than 0.05 and **P lower than 0.01 were considered statistically significant and are indicated versus the control unless noted otherwise.

## Results

### Crenolanib inhibits cell viability, cell migration and sprouting in vitro

**Fig. 1:**
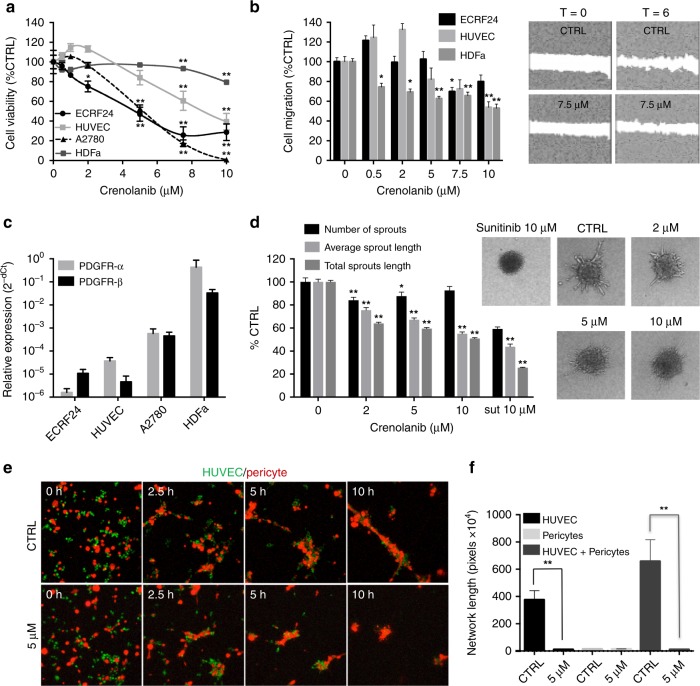
Activity of crenolanib on cell viability, migration and sprouting. **a** Cell viability dose response curves of crenolanib in endothelial cells (immortalised ECRF24 and primary human umbilical vein endothelial cells (HUVEC), ovarian cancer cells (A2780) and adult human dermal fibroblasts (HDFa). Cell viability was assessed after 72 h of exposure to crenolanib and represented as a percentage of untreated controls. Significance is indicated versus untreated cells. **b** Endothelial cell migration in response to crenolanib. Cell migration was assessed after 6 h drug treatment using a scratch assay. **c** PDGFR-α and -β expression in ECRF24, HUVEC, A2780 and HDFa determined by qPCR. **d** Activity of crenolanib on HUVEC sprouting. The number of sprouts and the average sprout length were quantified. **e** Representative images of HUVEC (green) and human pericyte (red) co-cultures. Co-cultures were established in 3D Matrigel matrices and allowed to randomly co-assemble over 10 h in the presence of DMSO or crenolanib (5 µM) at 0, 2.5, 5 and 10 h. **f** Quantification of the network length of capillary-like structures in HUVEC alone, pericyte alone and HUVEC/pericyte co-culture in the presence or absence of crenolanib (5 µM) at 10 h. All values shown are presented as percentage of the CTRL and represent the mean of at least two experiments performed in triplicate. Cells treated with 0.1% DMSO were used as a control (CTRL). Error bars indicate SEM. Significance (*P < 0.05, **P < 0.01) is indicated as compared to control

The activity of crenolanib was investigated in immortalised human endothelial cells (ECRF24), freshly isolated primary human umbilical vein endothelial cells (HUVEC), human ovarian carcinoma cells (A2780) and adult human dermal fibroblasts (HDFa). Cell viability was dose-dependently and significantly (ECRF24 2–10 µM; HUVEC 7.5–10 µM and A2780 5–10 µM) inhibited in ECRF24, HUVEC and A2780 cells after exposure to crenolanib for 72 h, with comparable IC_50_ values (i.e. 5.1 µM for A2780, 4.6 µM for ECRF24 and 8.4 µM for HUVEC, Fig. 1a). In contrast, crenolanib did not affect HDFa cell viability.

Cell migration, evaluated using the scratch assay, was significantly and dose-dependently inhibited in ECRF24, HUVEC and HDFa (Fig. 1b). Interestingly, crenolanib administered at lower doses (0.5–2 μM) tended to stimulate (not significantly) rather than inhibit EC migration, particularly in HUVEC (Fig. 1b). Of note, the inability of A2780 cells to form confluent monolayers precluded us to investigate this trait in these cells. Furthermore, we confirmed absence of viability inhibition during the time frame of the assay, indicating that a direct effect on cell migration was found (data no shown).

Strikingly, when we addressed the expression of the main targets of crenolanib, i.e. PDGFR-α and PDGFR-β, we noted that their expression was almost undetectable in A2780, ECRF24 and HUVEC (Fig. 1c and Supplementary Fig. 1), whereas HDFa showed marked expression. This seemingly counterintuitive observation urged us to further investigate the mode of action of crenolanib in these different cells.

In the next step, the activity of crenolanib was investigated in a collagen-based three-dimensional endothelial cell sprouting model (Fig. 1d). Average sprout length and total sprout length were decreased dose-dependently by crenolanib, whereas the number of sprouts was only minimally affected and decreased only at a dose of 2 µM (16 ± 2.7% as compared to CRTL). These results suggest that a reduced sprout length is due to inhibition of endothelial cell proliferation and sprout elongation. To further investigate this, we analysed the effect of crenolanib on the percentage of tip cells by flow cytometry using CD34 as a marker in HUVEC.^17,18^ Crenolanib administered at 5 μM for 72 h resulted in a significant increase in the number of CD34^+^ tip cells (Supplementary Fig. 2A). Next, to assess whether crenolanib acts differentially on CD34^+^ tip cells versus non-tip cells, we immunolabeled cells with antibodies for Annexin-V (for apoptotic cells) and CD34 (for tip cells) and analysed the cells by FACS. No differences were found in the percentage of apoptotic cells between CD34^+^ and CD34^-^ cells (Supplementary Fig. 2B).

To investigate further the anti-angiogenic mechanism of action of crenolanib, qPCR analysis was performed in HUVEC after crenolanib treatment (Supplementary Fig. 2C). A panel of tip cell genes was included in the analysis to test if crenolanib stimulates the expression of genes corresponding to the observed increase in tip cells. This panel included CD34, vascular endothelial cell growth factor receptors (VEGFR2–3), the endothelial cell Notch ligand Dll4, angiopoietin 2 (ANGPT2), CXC chemokine receptor 4 (CXCR4), netrin receptor UNC5B and insulin like growth factor 2 (IGF2).^18^ Administration of crenolanib at 5 μM in HUVEC resulted in an increase in mRNA levels of three out of eight tip cell specific genes, including CD34 mRNA. This indicates that the effect of crenolanib may not be tip cell specific. Expression of VEGFR2 and is significantly decreased as a result of crenolanib treatment, whereas VEGFR3 mRNA levels are not affected.

### Crenolanib regulates pericyte recruitment in vitro and affects cell morphology

In angiogenesis, endothelial cells of newly formed blood vessels produce PDGF to attract pericytes, a process that results in vessel stabilisation and maturation.^19^ In order to assess the role of crenolanib in pericyte recruitment, an in vitro endothelial–pericyte co-culture assay was applied to mimic pericyte recruitment and attachment to endothelial cells in vivo.^20,21^ Human pericytes (labelled with red cell tracker) were cultured with or without HUVEC (labelled with green cell tracker) on Matrigel (Fig. 1f and Supplementary Fig. 3). During a 10 h incubation, HUVEC alone aligned to form capillary-like cords in the control condition while they did not efficiently align in the presence of crenolanib (5 µM) (Suppl. Figure 3A and Supplementary Videos 1, 2). Pericytes alone were not able to align to form capillary-like cords in either control or crenolanib conditions (Supplementary Fig. 3B and Supplementary Videos 3, 4). Time-lapse imaging of capillary-like assembly revealed that after 10 h of co-culture, pericytes were tightly associated with endothelial cells in the control conditions (Fig. 1e and Supplementary Video 5). Strikingly, pericytes that were co-cultured with HUVEC in the presence of crenolanib did not closely associate and extended away from the cords in certain regions (Fig. 1e and Supplementary Video 6). Measurement of the length of the capillary-like networks confirmed the observed differences between cultures of HUVEC and/or pericytes in the presence or absence of crenolanib (Fig. 1f).

**Fig. 2:**
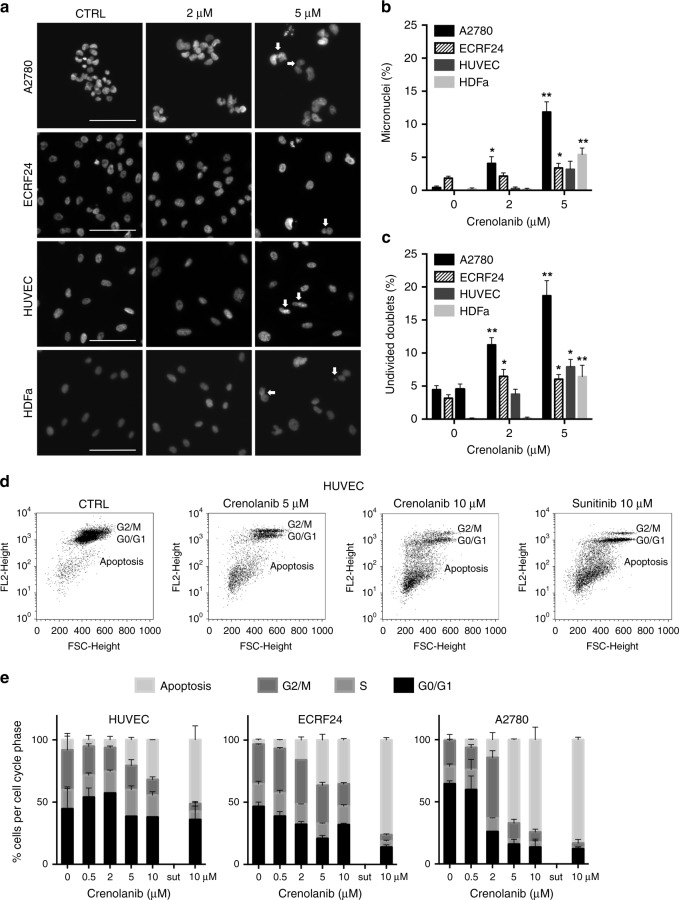
Crenolanib induces nuclear aberrations and apoptosis. **a** DAPI staining of A2780, ECRF24, HUVEC and HDFa after treatment with crenolanib for 72 h. In cells treated with crenolanib 5 µM white arrowheads indicate either micronuclei or undivided nuclear doublets. **b**, **c** Quantification of the percentage of micronuclei (**b**) and undivided nuclear doublets (**c**) based on images of DAPI staining. Values shown represent the percentage aberrations of the total amount of cell nuclei per image field. **d** Propidium-iodide staining of HUVEC treated with crenolanib for 72 h using flow cytometry. Cellular DNA content in permeabilised cells is proportional to fluorescence intensity (FL2-H; *y*-axis) and allows for the distinction of cells containing diploid DNA (G0/G1), tetraploid DNA (G2/M) and subdiploid DNA (apoptotic fraction). **e** Quantification of cellular DNA distribution over cell cycle phases as indicated in HUVEC, ECRF24 and A2780 cells after crenolanib treatment. All values shown are presented as percentage of the CTRL and represent the mean of at least two experiments. Error bars indicate SEM. Significance (*P < 0.05, **P < 0.01) is indicated as compared to control

To further investigate the cellular morphology and integrity in response to crenolanib treatment, ECRF24, HUVEC, A2780 and HDFa were stained for F-actin (phalloidin) and DNA (DAPI; Supplementary Fig. 4A). The actin cytoskeleton consists of a membrane supporting component, a cortical actin rim and actomyosin based stress fibres.^22^ In non-treated cells, especially in ECRF24 and HUVEC, the stress fibres and cortical rim can clearly be observed. Treatment with crenolanib 5 µM results in cell border retraction and gap formation. In all cell lines, treatment with increasing crenolanib dose led to the formation of micronuclei which may be indicative of genomic instability and chromosomal damage (Fig. 2a).^23^ In addition, undivided cell nucleus doublets can be observed that may imply a halt in cell division and thus cell proliferation (Fig. 2a). Quantification revealed a significant increase of the total number of micronuclei and undivided cell nucleus doublets (Fig. 2b, c). Interestingly, this result was most profound in A2780 cells.

Next, HUVEC were also stained for the adherens junction molecule VE-cadherin (VE-cad) and DNA (DAPI). Treatment with crenolanib resulted in a decrease of adherens junctions that have a ruffled appearance and formation of intercellular gaps (indicated by white arrowheads; Supplementary Fig. 4B) in accordance to the activity observed in F-actin staining that suggests cell border retraction (Supplementary Fig. 4A).

### Crenolanib induces apoptosis in both endothelial and ovarian cancer cells before mitotic entry

To study the mechanism of crenolanib-induced suppression of cell viability in EC and ovarian cancer cells, DNA profiles of crenolanib treated cells were assessed by flow cytometry analysis of sub-diploid cells, after staining with propidium iodide. Sunitinib (10 μM) was used as a positive control in these assays.^11^ Crenolanib-induced apoptosis in ECRF24, HUVEC and A2780 (Fig. 2d, e and Supplementary Fig. 5). Ovarian cancer cells were more sensitive to apoptosis induction by crenolanib than EC.

**Fig. 3:**
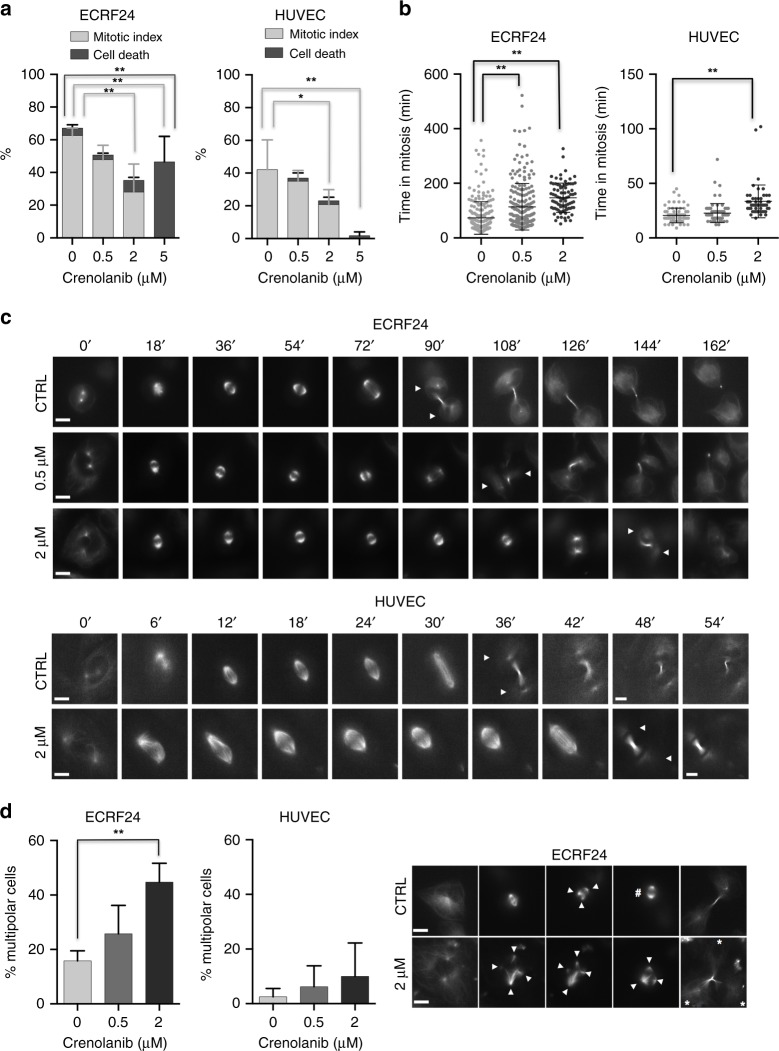
Crenolanib impairs mitosis and prevents centrosome clustering. **a** Percentage of ECRF24 and HUVEC cells entering mitosis during the 24 hr live-cell imaging movies (mitotic index) and the percentage of cell death after treatment with 0.5, 2 and 5 µM of crenolanib or DMSO. **b** Plots for the timing between nuclear envelope breakdown and anaphase onset in minutes (mitotic timing). Number of cells are ECRF24: 0 µM *n* = 230, 0.5 µM *n* = 229, 2 µM *n* = 84; HUVEC: 0 µM *n* = 81, 0.5 µM *n* = 80, 2 µM *n* = 54. **c** Representative live-cell imaging stills over time of mitotic ECRF24 (top) and HUVEC cells stained with SiR-tubulin (microtubule marker) and treated with indicated doses of crenolanib. The arrows indicate the two daughter cells. **d** Quantification of multipolar spindles in mitosis in ECRF24 and HUVEC cells treated with indicated crenolanib concentrations. On the right, representative images of a mitotic ECRF24 cell (CTRL and crenolanib 2 µM) displaying multipolar spindles (indicated by arrows) are shown. The hashtag symbol indicates the centrosome clustering event and the asterisks the daughter cells resulting from the asymmetric division. Scale bars indicate 10 µm. Significance (*P < 0.05, **P < 0.01) is indicated as compared to control (0.1% DMSO) and error bars indicate SD

To investigate in more detail how crenolanib affects cell proliferation and induces apoptosis in endothelial cells, we performed live-cell imaging of ECRF24 and HUVEC cells treated with increasing doses of crenolanib. To monitor mitotic events in particular, and the behaviour of the mitotic spindle, cells were stained with the live-cell dye SiR-tubulin that stains microtubules.^24^ Our analysis revealed that crenolanib affected the cell cycle and mitosis at several levels. First, crenolanib prevented mitotic entry, as with increasing doses a smaller percentage of ECRF24 and HUVEC entered mitosis over the period of 24 h (Fig. 3a). In particular, at 5 µM crenolanib, mitotic entry was completely blocked in both cell types. Second, crenolanib significantly increased the duration between mitotic entry and mitotic exit (mitotic timing) in both cell types (Fig. 3b, c). Since mitotic timing is mostly determined by the ability of cells to attach all chromosomes on kinetochores and satisfy the spindle assembly checkpoint,^25^ this implied that crenolanib partially impairs chromosome attachment by the mitotic spindle. Third, crenolanib has been previously reported to prevent the clustering of spindle poles (centrosome clustering) in cancer cells in the presence of multipolar spindles.^26^ Consistent with this study, 2 µM crenolanib prevented centrosome clustering in ECRF24 cells, as we observed an increased percentage of mitotic cells with multipolar spindles; HUVEC cells had much fewer multipolar spindles to start with and we did not see a significant increase after crenolanib treatment (Fig. 3d). Finally, despite these mitotic defects, we observed that crenolanib induced cell death mostly independently of mitosis, as the occurrence of cell death did not correlate with any mitotic outcome. In particular, 5 µM crenolanib-induced massive cell death in ECRF24 cells (0 µM = 4.5% ± 2.0 vs. 5 µM = 46.5% ± 15.6%) despite the absence of mitosis (Fig. 3a). We further note that in this assay, HUVEC cells appeared to be less sensitive to crenolanib-induced cell-death (Fig. 3a).

Taken together, we show that crenolanib induces apoptosis at increasing dose levels. Furthermore, despite partial impairment of mitotic progression, crenolanib mostly affects ECRF24 and HUVEC cells before cell division by preventing mitotic entry and inducing a cell division-independent cell death.

### Crenolanib inhibits tumour growth, microvessel density and vascular sprouting in the CAM model

**Fig. 4:**
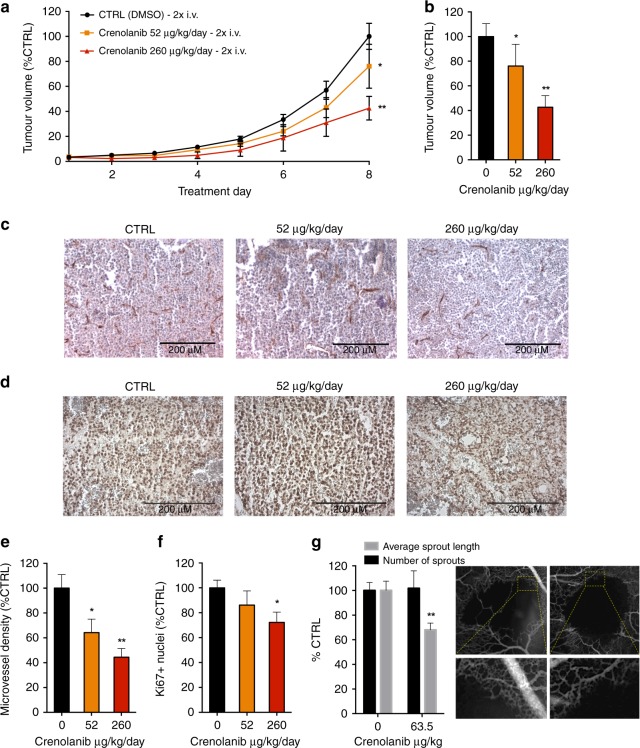
Crenolanib inhibits A2780 tumour growth and PDT-induced sprouting in the CAM model. **a** Tumour growth curves of A2780 spheroids xenografted on the CAM. Tumour volume is represented as the percentage of control tumour volume in respect to treatment days. *n* = 41 in the control group and *n* = 12–27 in the treatment groups. Eggs were treated with vehicle (0.1% DMSO) or crenolanib (52 µg/kg/day and 260 µg/kg/day) 2x daily i.v. injections. **b** Final tumour volume at the day of tumour resection. **c** Representative images of tumour sections stained for CD31. **d** Representative images of tumour sections stained for Ki67. **e** Microvessel density analysis of tumour sections stained for the endothelial cell marker CD31. **f** Analysis of the fraction of Ki67 positive cell nuclei presented as the percentage of stained cells per image field. **g** Effect of crenolanib at a dose of 63.5 µg/kg on PDT-induced vascular sprouting in the CAM. Quantification of the number of sprouts and the average sprout length are shown as percentage of control. Significance (*P < 0.05, **P < 0.01) is indicated as compared to control (0.1% DMSO treated cells) and error bars indicate SEM. Representative images 24 h after PDT are shown. The bottom panel indicates zoomed areas corresponding to yellow boxes in the upper panel

Finally, the vascular and anti-tumour activity of crenolanib was investigated in the CAM model. First, the activity of crenolanib was assessed on human A2780 ovarian carcinoma grown on the CAM. Crenolanib treatment was performed once daily for two days (EDD 11–12) at doses of 52 μg/kg/day and 260 μg/kg/day (corresponding to 5 μM and 25 μM of crenolanib in 100 μL saline, respectively). Daily injections were performed mimicking the clinical use of crenolanib considering its short in vivo half-life of 8 h.^27^ A 2-day treatment of i.v. administration of crenolanib resulted in significant tumour growth inhibition up to 57.4 ± 9.4% (260 μg/kg/day; Fig. 4a, b).

During tumour growth of A2780 ovarian carcinoma on the CAM, the endothelial mRNA levels of PDGFR-α and β were considerably increased (Supplementary Fig. 6), consistent with ongoing angiogenesis. To investigate the effect of crenolanib treatment on the tumour vasculature, microvessel density (MVD) was quantified by staining for the endothelial cell marker CD31 in tumour sections (Fig. 4e). Administration of crenolanib (260 μg/kg/day) resulted in a significant decrease in MVD up to 55.6 ± 7.0%. Additionally, tumour sections were also stained for the proliferation marker Ki67. Quantification revealed a significant decrease in the amount of Ki67 positive cell nuclei in crenolanib (260 μg/kg/day) treated tumours (Fig. 4f).

Next, in vivo vascular sprouting was assessed following vaso-occlusive Visudyne^TM^-PDT.^14^ Application of PDT to the CAM vasculature leads to occlusion of blood vessels, which subsequently induces an angiogenic switch leading to the revascularisation of the treated CAM areas.^14^ The administration of crenolanib directly after PDT on EDD 11 resulted in a decrease of the average sprout length, but not the number of sprouts, 24 h after PDT as compared to the control (Fig. 4g). Strikingly, this result strongly resembles the effect of crenolanib seen in EC sprouting in vitro where the number of sprouts was not affected in contrast to the average sprout length (Fig. 1c).

In summary, crenolanib inhibits tumour growth in a dose-dependent manner, which, at least in part, is caused by MVD inhibition. Furthermore, a decrease in the length of vascular sprouts is observed while the number of sprouts is not affected. These results in the CAM model confirm the strong anti-angiogenic activity observed in previous experiments.

### In silico analysis of alternative mechanism of action of crenolanib

**Fig. 5:**
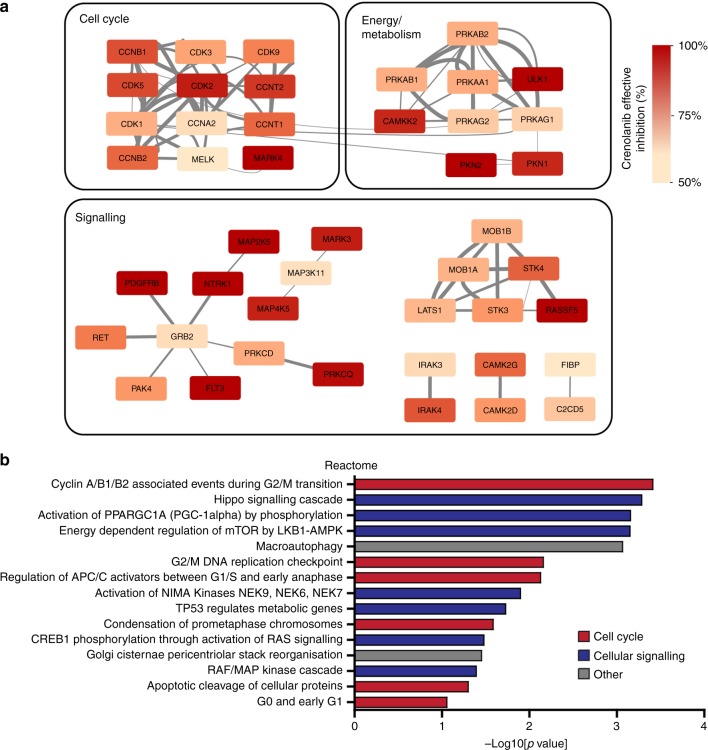
In silico analysis of crenolanib target proteins. Crenolanib target proteins with an effective inhibition (EI%) of ≥50% with 5 μM crenolanib were retrieved from proteomicsDB (https://www.proteomicsdb.org) and subject to network analysis via STRING (**a**) and enrichment analysis using DAVID (**b**) as described in materials and methods. **a** Protein-protein interaction data from STRING were visualised in Cytoscape where the node colours reflect the EI% and the thickness of the edges indicate confidence levels of the interaction. **b** Enrichment of functional clusters for the Reactome database

In order to explain the observed phenotypic effects of crenolanib in cells that do not express the target receptors, we mined ProteomicsDB repository for additional protein targets of crenolanib. Using a drug concentration of 5 μM and an effective inhibition score of ≥50%, 63 proteins were retrieved. Pathway enrichment analysis of these proteins clearly demonstrated their involvement in cell division organisation (Fig. 5a and Supplementary Fig 7). Furthermore, protein interaction analysis using STRING identified tight clustering and multi-level interactions between cell cycle related proteins, whereas the main targets of crenolanib (i.e. PDGFR-β and FLT3) are present in an unconnected cluster (Fig. 5b). As such, these data support and explain our observations on the predominant effects on mitosis and apoptosis by crenolanib.

## Discussion

Angiogenesis inhibition currently is a widely used treatment strategy for patients with cancer. Although many angiogenesis-targeted drugs are limited in their ability to prolong overall patient survival, many novel therapeutic targets and combination-based strategies are being developed with the aim of improving angiogenesis suppression and overcoming limitations associated with angiogenesis inhibition, such as toxicity and resistance.^28,29^

The results presented in this study show that crenolanib exerts strong anti-angiogenic activity in both in vitro and in vivo angiogenesis models, as well as direct anti-cancer activity. Analysis of PDGFR expression in the included cell lines (ECRF24, HUVEC, A2780) revealed that PDGFR expression barely exceeded detection levels as compared to positive control cell line HDFa. Thus, crenolanib appears to act on both endothelial cells and cancer cells independent of PDGFR expression. It should be noted that crenolanib is a type I inhibitor which means the drug only acts on active receptor conformations (i.e. binding of PDGF to its receptor). Consequently, PDGF ligand must be present for crenolanib to exert its effect. In our assays, however, we did not evaluate conditions with excess PDGF ligand.

Since the activity of crenolanib cannot be explained as a result of PDGFR receptor targeting in EC, it is likely that there are other mechanisms at play that account for the activity of crenolanib. First, we showed that crenolanib reduces cell viability and cell migration of HUVEC and ECRF24 cells in a dose-dependent manner (Fig. 1a, b). Studying the effect of crenolanib on HUVEC sprouting in a 3D collagen-based model revealed that only the sprout length was affected and not the number of sprouts (Fig. 1d). This was confirmed by analysis of vascular sprouting in the CAM model after PDT-induced angiogenesis (Fig. 4g). In our previous work we have demonstrated that the PDT CAM angiogenesis is much more sensitive (10-fold as compared to the angiogenesis inhibitors used in the cited study) than the developmental CAM model and resembles better the tumour microenvironment.^14^

The observation that EC viability, migration and sprout length, but not sprout initiation was inhibited prompted us to study variable activity of crenolanib on different EC phenotypes. Specifically, ECs can differentiate into tip cells and non-tip cells (mainly existing of stalk cells, but also phalanx cells and quiescent cells). The EC tip cell phenotype is characterised by long filopodia and migratory activity whereas the stalk cell phenotype is characterised by proliferative activity and sprout stabilisation.^30^ Thus, the strong anti-proliferative effect of crenolanib may be primarily due to activity on the proliferative stalk cells, accounting for a significant decrease in sprout length without a reduction in the overall number of sprouts.^17^

Studying cell morphology in response to crenolanib revealed that crenolanib significantly affects the actin cytoskeleton resulting in cell contraction and ultimately gap formation (Supplementary Fig. 4a). Staining for adherens junction molecule VE-cadherin revealed a decrease and loosening of adherens junctions which may contribute to the observed gap formation (Supplementary Fig. 4b). Both a loss of adherens junctions and actin cytoskeleton structure may lead to increased vascular permeability.^31^ Furthermore, after visualisation of cell nuclei with DAPI we observed the appearance of micronuclei and undivided doublets. The appearance of micronuclei may be indicative of genomic instability, chromosomal damage and apoptosis (Fig. 2b, c).^23^ We described this phenomenon previously as a result of an angiostatic combination therapy.^32^

Due to the observation of micronuclei and undivided doublets we hypothesised that crenolanib may halt or affect mitosis. Crenolanib was found to increase mitotic timing in ECRF24 and HUVEC and to prevent centrosome clustering in ECRF24 cells (Fig. 3). Crenolanib was previously described to prevent centrosome clustering in cancer cells but this has not been described for other cell types.^26^ The authors demonstrated that crenolanib induced ‘activation of the actin-severing protein cofilin, leading to destabilisation of the cortical actin network’ and found that this activity was unrelated to PDGFR-β expression.^26^ Nevertheless, here we found that crenolanib mostly affected cell viability and cell proliferation of endothelial cells before or independently of cell division, as we observed a dose-dependent block in mitotic entry, and an increase in apoptosis independent of mitotic events.

To further assess the mechanism of action of crenolanib we searched for crenolanib target proteins in an online proteomics database and found a total of 63 proteins (Supplementary Table 1) that were reported to have an effective inhibition of ≥50% by 5 μM of crenolanib. Subsequent gene ontology analysis showed considerable enrichment for cell cycle and cell division related proteins, in line with the results presented here (Fig. 5b and Supplementary Fig. 7). Various proteins of the Hippo pathway were also identified as targets of crenolanib. Together, these data point to a more complex mechanism of action of crenolanib that is not only mediated by inhibition of PDGFR and FLT3, but which is also heavily relying on disruption of normal cellular proliferation.

The anti-angiogenic activity of crenolanib observed in various in vitro assays was confirmed in vivo in tumours grown on the CAM. Here we report that crenolanib inhibits tumour growth at doses of 52 μg/kg/day and 260 μg/kg/day (Fig. 4a, b). Tumour growth inhibition was found to correlate to a decrease in MVD based on CD31 staining (Fig. 4e) and to a decrease in the density of proliferating cells, assessed by quantification of Ki67 staining (Fig. 4f).

In summary, we present a thorough analysis of the anti-angiogenic activity of the crenolanib. Since the effects of crenolanib presented in this study appear unrelated to PDGFR expression, we propose several other mechanisms that account for the observed activity. Based on our findings, we propose crenolanib for further investigations in other solid tumour types and clinical evaluation in patients with solid tumours.

## Supplementary information

Supplementary Information

Video 1

Video 2

Video 3

Video 4

Video 5

Video 6

## Data Availability

All the data generated or analysed in this study are included in the manuscript or the supplementary information.
