# Supplementary material for: Anti-angiogenic effects of crenolanib are mediated by mitotic modulation independently of PDGFR expression
Source: Br J Cancer. 2019 Jun 25;121(2):139–49. doi: 10.1038/s41416-019-0498-2 (PMC6738084; doi:10.1038/s41416-019-0498-2)
Supplement: Supplementary file 1 — Supplementary Information [file 41416_2019_498_MOESM1_ESM.docx]

**Berndsen *et al*. Supplementary Information**

**Supplementary Figures**

**Supplementary Videos**

**Supplementary Methods**

**Supplementary Tables**

**Supplementary References**

**Supplementary Figures**

**Supplementary Figure 1**. Western Blot and flow cytometry analysis of PDGFR-β in ECRF24, HUVEC, A2780 and HDFa. (A) Western Blot analysis showing PDGFR-β expression at 190 kDa in HDFa only. Beta-actin loading control (42 kDa) was included for all conditions. (B) Quantification of the mean fluorescence intensity of cells expressing PDGFR-β compared to a negative control. Significance is indicated versus PDGFR-β fluorescence intensity in HDFa cells. The values shown represent the mean of at least two independent experiments and error bars indicate the SEM. (C) Representative FACS plots showing the shift of fluorescence intensity in the FL1-H channel based on immunolabeling of PDGFR-β.

**
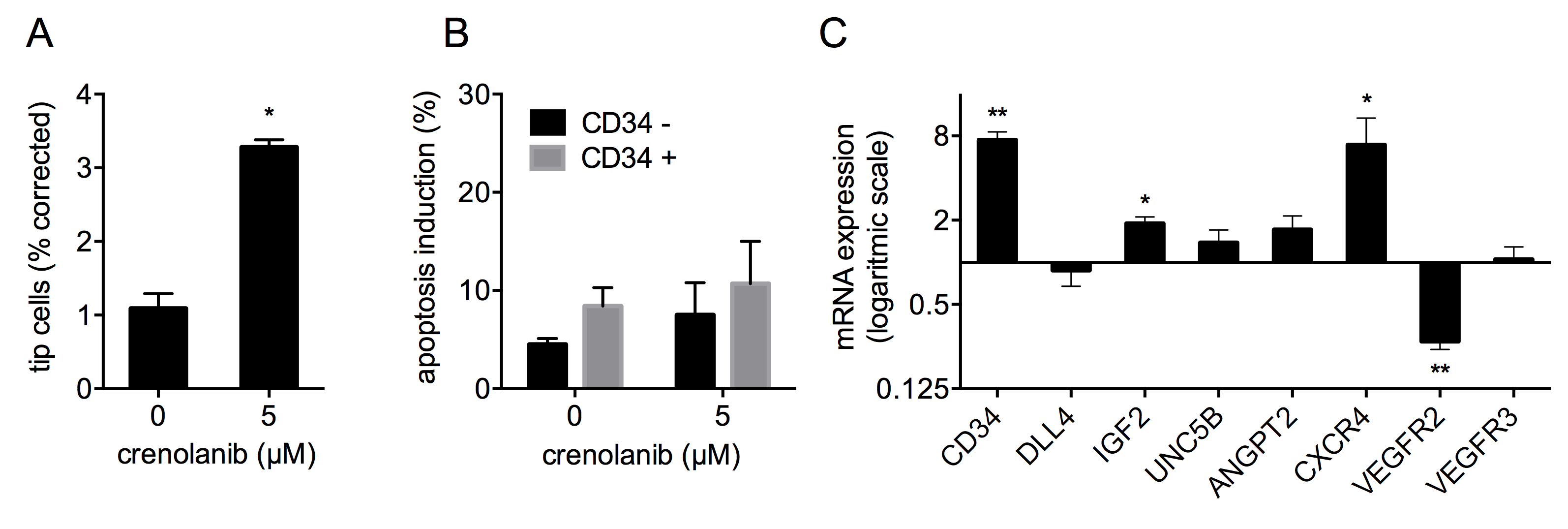
**

**Supplementary Figure 2.** Differential activity of crenolanib on specialized HUVEC phenotypes. (A) Percentage of CD34+ cells in response to crenolanib treatment. (B) Apoptosis induction in CD34+ and CD34- cells. HUVEC were double-labeled with anti-CD34 and anti-Annexin-V antibodies for dual color flow cytometry to determine the fraction of (early) apoptotic cells (Annexin-V+) in the fractions of CD34+ (tip cells) and CD34- (non-tip cells). (C) Effect of crenolanib (5 µM) on mRNA levels of tip cell specific genes in HUVEC. Values shown represent fold-change compared to CTRL mRNA expression (untreated cells) represented as mean 2^dCt values. For all panels, the values shown represent the mean of at least two independent experiments and error bars indicate the SEM. *p < 0.05, **p < 0.01.

**Supplementary Figure 3.** Network formation of crenolanib- or non-treated (A) HUVEC (green labelled cells) and (B) pericytes (red labelled cells). Representative images are shown. Cultures were established in 3D Matrigel matrices and allowed to randomly co-assemble over 10 hours. Scale bars represent 200 µm. Quantification of network formation and images of HUVEC/pericyte co-cultures are presented in Figure 1 F.

**Supplementary Figure 4.** Effect of crenolanib on cell morphology. (A) DAPI (blue) and F-actin (phalloidin; green) immunofluorescence staining of A2780, ECRF24, HUVEC and HDFa exposed to 0, 2 and 5 μM crenolanib. (B) DAPI (blue) and VE-Cadherin (green) immunofluorescence staining of HUVEC exposed to 0, 1 and 5 μM crenolanib. White triangles indicate gap formation in cultures treated with crenolanib 5 μM. Cells were treated for 72 hours.

**Supplementary Figure 5.** Representative FL2-H/FSC-H FACS plots of (A) ECRF24 and (B) A2780 stained for DNA content with propidium iodide (PI). Sub-diploid cells, characterized by a decrease in fluorescently stained nuclear DNA, were gated as apoptotic cells. CTRL cells were treated with 0.1% DMSO and sunitinib 10 μM was used as positive control for apoptosis induction.

**Supplementary Figure 6.** Relative expression levels of PDGFR-α and β in naïve CAMs and in CAMs xenografted with A2780 tumors. For reference and presentation, the expression of PDGFR-α was set to 1 in CAM.

**
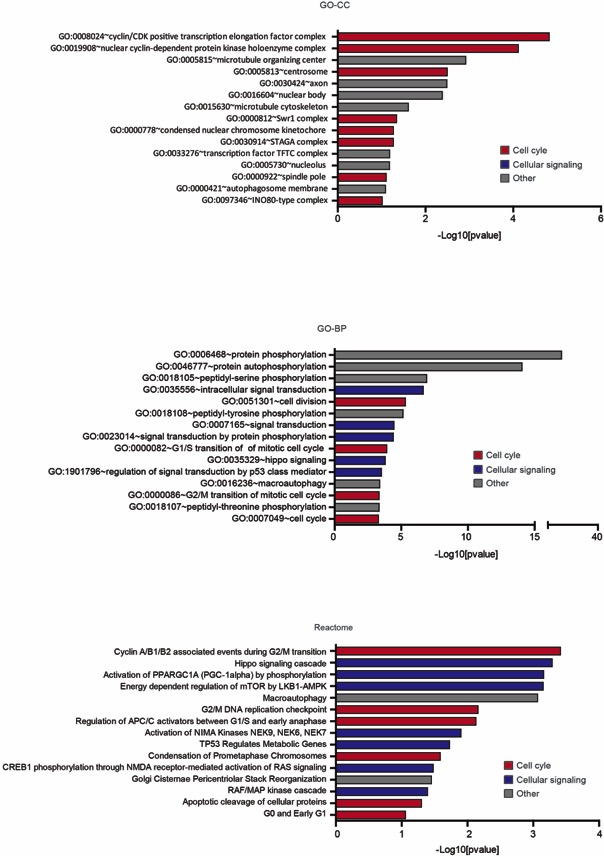
**

**Supplementary Figure 7**. *In silico* analysis of crenolanib target proteins. Functional annotations were retrieved using DAVID and mined for enrichment in gene ontologies (GO-CC: Cellular component; GO-BP: biological process.

**Supplementary Videos**

**Supplementary Videos 1-6.** Fluorescent time-lapse microscopy of pericyte recruitment to endothelial cell tubes (HUVEC). HUVEC (green) and human brain pericytes (green) were established as co-culture in 3D Matrigel in the presence of DMSO (0.024%) and crenolanib (5 µM) and then imaged by time-lapse microscopy for the next 10 hours. Frames were collected every hour and are shown at 10 frames/sec.

**Supplementary Methods**

*Preparation of collagen gel mixture for sprouting assay*

Collagen gel mixture was prepared by adding a solution A to a solution B. Solution A contained (in % of total volume): 66.66% PureCol^®^ (Sigma-Aldrich), 8.34% 10x M199 medium (Sigma-Aldrich) and 0.2 M NaOH while shaking until a color shift of the solution to red/purple was seen. Solution B contained (in % of total volume): 10% new born calf serum (NBCS; Sigma-Aldrich), 0.1% heparin and a mixture of 14.9% methylcellulose/RPMI medium. Solution A and B were then mixed and bFGF was added to a final concentration of 50 ng/ml.

*RNA isolation and quantitative PCR*

Total RNA was isolated from HUVEC using the TRIzol method according to the manufacturer’s instructions (Invitrogen, Carlsbad, CA, USA). 1 μg RNA was used for DNase I treatment (amplification grade; Invitrogen) and reverse transcribed into cDNA using the Maxima First Strand cDNA Synthesis Kit (Thermo Scientific) or iScript cDNA synthesis kit (Bio-Rad). Primers were designed and validated as previously described (Suppl. Table 2)^3^. qPCR was performed using a CFX96 real-time PCR detection system (Bio-Rad Laboratories, Hercules, CA, USA) using SYBR green.^3^ Relative expression of PDGFRs in the cell lines under study (HDFa, A2780, ECRF24, HUVEC), as well as for the CAM was expressed using the 2^-dCt method relative to the average expression of cyclophillin A and b-actin. For the tip cell gene panel in HUVEC, gene expression data was normalized to tyrosine 3-monooxygenase/tryptophan 5-monooxygenase activation protein and zeta polypeptide (YHWAZ) levels and by the factor correction method for between-donor variation.^4^

*Flow cytometry*

PDGFR-β expression was determined by flow cytometry. Following harvesting by trypsin, cells were fixated in 70% ethanol for >2hr at -20ºC. Cells were washed with PBS, blocked in 1% BSA in PBS for 30 minutes at room temperature and subsequently incubated with rabbit anti-PDGFRB antibody (Clone 28E1, Cell Signaling Technologies, 1:50), followed by biotinylated swine anti-rabbit IgG (Dako) and streptavidin-Alexa488 (Life Technologies), separated by washes in PBS. Cells were analysed on a FACSCalibur (BD Biosciences) and data were analysed using CellQuest software (BD Biosciences). Mean fluorescence intensities were reported as percentage of negative controls, averaged over 3 independent cell isolates.

*HUVEC maintenance in tip cell studies*

Primary HUVECs were isolated from umbilical cords, as described earlier,^1^ and grown in M199 medium (Gibco, Grand Island, NY, USA) supplemented with 10% heat-inactivated human serum (HS), 10% fetal bovine serum (Gibco), and 1% penicillin-streptomycin-glutamine (Gibco). HUVECs were cultured in T75 culture flasks coated with 2% gelatin (Millipore, Billerica, MA, USA) at 37°C and 5% CO_2_. Experiments were performed with confluent HUVEC passage 3-4 cells of at least 3 different donors. Subjects gave informed consent for the use of tissues or serum and samples were stored anonymously.

*Endothelial-Pericyte co-culture network formation assay*

The assay was adapted from Tigges, *et al.* (2012).^2^ Briefly, adherent HUVEC and human pericytes were labeled with 1 μM CellTracker Orange CMRA548 (Thermo Fisher Scientific) and 1 μM CellTracker Green CMFDA488 (Thermo Fisher Scientific) dyes, respectively, in serum-free M199 medium for 30 minutes at 37°C. First, 10-12 μL of ice-cold growth factor-reduced Matrigel (Corning) was added into the wells of a 15-well angiogenesis micro-slide (Ibidi) and allowed to polymerize. Second, 50 μL of complete M199 medium was added to each well and incubated for 45 minutes at 37°C. Both HUVEC and pericytes were then harvested by trypsinization and counted. Approximately, 2.500 HUVEC and 5.000 pericytes were added to the polymerized Matrigel in each well, and cultured in complete M199 for up to 10h. The co-cultures were analyzed by live-cell time-lapse imaging using Nikon A1R confocal microscope with the piezoelectric objective drive allowing rapid collection of Z-series images (Nikon). The live-cell z-series imaging for 60 min periods was acquired by NIS Elements software (Nikon) and conducted in a live imaging chamber supplied with 5% CO_2_ at 37°C. The lasers and emission band passes used for imaging were as follows: green, excitation: 488 nm, emission: 500-550 nm; red, excitation: 561 nm, emission: 570-620 nm. For generating videos, maximum projections of the z-series were collected and time-lapse videos were generated and analyzed from the acquired images at a frame rate of 10 fps using Fiji/Image J with Angiogenesis Analyzer toolset (NIH).

*Immunofluorescence assay*

Cells were fixed in freshly-made 4% paraformaldehyde in PBS for 15 min at room temperature, and then blocked in PBS containing 10% bovine serum albumin (Sigma-Aldrich, St. Louis, MO, USA) and 0.5% Triton X-100 (Sigma). VE-cadherin and cell nuclei were visualized by a combination of rabbit-anti VE-cadherin (cat. ab33168; 1:400, Abcam, Cambridge, UK) and DAPI (D9542, Sigma-Aldrich, St. Louis, MO, USA).

*Flow cytometry analysis of apoptosis in tip cells and non-tip cells*

To compare apoptotic fractions in tip cells versus non-tip cells, cells were stained for the tip cell marker CD34 and Annexin V for apoptotic cells. Cells were fixed in 2% paraformaldehyde in PBS for 15 min at room temperature and incubated with anti-CD34-phycoerythrin antibody (anti-CD34-PE; clone QBend-10, Thermo Scientific, Waltham, MA, USA) without any permeabilization of the cells to measure CD34 on the cell surface only. Cells were analyzed by flow cytometry using a FACSCalibur (BD Biosciences, Franklin Lakes, NJ, USA) and FlowJo 6.4.7 software (Tree Star, San Carlos, CA, USA). Non-stained, non-treated cells were used as negative controls. Annexin V-FITC labeling was performed according to manufacturer’s instructions (Molecular Probes, Eugene, OR, USA).

*Immunohistochemistry*

Briefly, tumors were fixed overnight in zinc fixative solution, embedded in paraffin and 5 µm sections were prepared. Sections were incubated in methanol containing 0.3% H_2_O_2_, boiled in citrate buffer (10mM, pH 6) and blocked with 5% bovine serum albumin (BSA) in PBS. Subsequently, tumor sections were incubated with primary antibodies against CD31 (1:200; clone SZ31, Dianova, Hamburg, Germany). Secondary donkey anti-rat biotinylated antibodies (1:200; Jackson, Suffolk, UK) were then incubated, followed by streptavidin-HRP (1:50; Dako, Glostrup, Denmark) and visualized by 3,3'-diaminobenzidine (DAB), resulting in a brown-colored precipitate at the antigen site. Sections were stained separately for proliferation marker with primary antibodies against Ki67 (rabbit anti-human Ki67, 1:100, clone SP6; Thermo Scientific) and subsequent incubation with secondary swine anti-rabbit biotinylated antibodies (1:200; Dako, Glostrup, Denmark). Ki67^+^ nuclei were visualized by DAB following streptavidin-HRP staining.

*In silico analysis of crenolanib target proteins*

Functional annotations were retrieved using DAVID (<https://david.ncifcrf.gov/>) and enrichment was analysed for diverse ontologies. Protein interaction clusters were visualised using String (<https://string-db.org/>). Interaction score cut-off was set to high confidence (0.9), and K-means clustering for 3 clusters was performed. For clarity, unconnected nodes were left out of the network graph. Data were exported to Cytoscape 3.7.1 where the nodes in the network were color coded along the effective inhibition score obtained from proteomicsDB, whereas the thickness of the edges reflects the connectivity.

*Primer sequences quantitative PCR*

Primer sequences for quantitative PCR experiments are listed in **Supplementary Table 1**.

**Supplementary Tables**

**Supplementary Table 1.** Crenolanib target proteins identified from an online proteomics database (<https://www.proteomicsdb.org>).

| **Gene Name** | **Unique Identifier** | **Crenolanib effective Inhibition [%]** | **Crenolanib EC50 [nM]** | |
| --- | --- | --- | --- | --- |
| CMPK1 | P30085 | 100 | 2,9 |  |
| **FLT3** | **P36888** | **100** | **32,9** |  |
| MAP2K5 | Q13163 | 100 | 47,4 |  |
| MARK4 | Q96L34 | 100 | 30,0 |  |
| MST4 | Q9P289 | 100 | 14,3 |  |
| NQO2 | P16083 | 100 | 40,1 |  |
| NTRK1 | P04629 | 100 | 12,0 |  |
| **PDGFRB** | **P09619** | **100** | **121,0** |  |
| PKN2 | Q16513 | 100 | 1488,8 |  |
| RASSF5 | Q8WWW0 | 100 | 191,5 |  |
| SIK3 | Q9Y2K2 | 100 | 2,9 |  |
| STK24 | Q9Y6E0 | 100 | 56,9 |  |
| TRRAP | Q9Y4A5 | 100 | 21,3 |  |
| ULK1 | O75385 | 100 | 1256,3 |  |
| SIK2 | Q9H0K1 | 99 | 31,1 |  |
| PRKCQ | Q04759 | 98 | 3063,3 |  |
| DCAF7 | P61962 | 97 | 997,0 |  |
| GARS | P41250 | 97 | 40,3 |  |
| CDK2 | P24941 | 95 | 239,8 |  |
| MARK3 | P27448 | 95 | 285,7 |  |
| MAP4K5 | Q9Y4K4 | 94 | 610,9 |  |
| CAMKK2 | Q96RR4 | 93 | 203,4 |  |
| MARK2 | Q7KZI7 | 93 | 398,7 |  |
| PKN1 | Q16512 | 93 | 189,3 |  |
| MAP4K4 | O95819 | 91 | 282,1 |  |
| CCNT2 | O60583 | 90 | 757,1 |  |
| CDK5 | Q00535 | 87 | 598,6 |  |
| CCNB1 | P14635 | 86 | 498,0 |  |
| IRAK4 | Q9NWZ3 | 85 | 1004,9 |  |
| STK4 | Q13043 | 83 | 920,7 |  |
| CAMK2G | Q13555 | 82 | 3524,7 |  |
| CCNB2 | O95067 | 82 | 932,4 |  |
| CCNT1 | O60563 | 82 | 1616,7 |  |
| MAP4K3 | Q8IVH8 | 79 | 619,1 |  |
| RET | P07949 | 78 | 1839,6 |  |
| CDK9 | P50750 | 77 | 1388,2 |  |
| BMP2K | Q9NSY1 | 74 | 1816,1 |  |
| STK10 | O94804 | 73 | 3745,1 |  |
| CAMK2D | Q13557 | 72 | 4027,3 |  |
| HSP90AB2 | Q58FF8 | 72 | 2565,8 |  |
| PAK4 | O96013 | 72 | 1676,0 |  |
| STK3 | Q13188 | 72 | 972,2 |  |
| CDK3 | Q00526 | 69 | 1065,5 |  |
| PRKAA1 | Q13131 | 67 | 1955,8 |  |
| PRKCD | Q05655 | 67 | 3155,9 |  |
| CDK1 | P06493 | 66 | 2008,2 |  |
| PRKAB1 | Q9Y478 | 66 | 1220,0 |  |
| PRKAB2 | O43741 | 66 | 1414,4 |  |
| MOB1A | Q9H8S9 | 65 | 2411,2 |  |
| MOB1B | Q7L9L4 | 65 | 2411,2 |  |
| LATS1 | O95835 | 64 | 3514,3 |  |
| GAK | O14976 | 60 | 4285,7 |  |
| KIAA0528 | Q86YS7 | 60 | 4177,8 |  |
| PRKAG2 | Q9UGJ0 | 59 | 2748,6 |  |
| PRKAG1 | P54619 | 57 | 3154,3 |  |
| IRAK3 | Q9Y616 | 55 | 3510,6 |  |
| CCNA2 | P20248 | 54 | 4167,8 |  |
| GRB2 | P62993 | 54 | 4209,9 |  |
| MAP3K11 | Q16584 | 53 | 4402,4 |  |
| RIPK2 | O43353 | 52 | 4676,9 |  |
| RPS6KA6 | Q9UK32 | 52 | 4696,5 |  |
| FIBP | O43427 | 51 | 4870,6 |  |
| MELK | Q14680 | 51 | 4783,0 |  |
| DYRK1A | Q13627 | 49 | 5322,5 |  |
| PHKG2 | P15735 | 49 | 5172,2 |  |
| FECH | P22830 | 34 | 7391,7 |  |
| PRKD3 | O94806 | 28 | 19950,8 |  |
| PDPK1 | O15530 | 1 | 12601,7 |  |
| PDPK2 | Q6A1A2 | 1 | 12601,7 |  |
|  |  |  |  |  |

**Supplementary Table 2.** Primer sequences for qPCR

| Gene (human) | Fw | Rv |
| --- | --- | --- |
| IGF2 | CCTCGTGCTGCATTGCTGCT | CTTGCGGGCCTGCTGAAGTAGAA |
| ANGPT2 | GCAAAATAAGCAGCATCAGCCAAC | GCATCAAACCACCAGCCTCCT |
| CD34 | GCCAGGTATTACAACTGGGTGTCCT | TCTGGTTGTCCACAGAGCCTTTGT |
| CXCR4 | AACGGGGGACAGTGCCTGAA | TGAGCCCATTCTCCAGGTCAT |
| DLL4 | AACGGGGGACAGTGCCTGAA | TGAGCCCATTCTCCAGGTCAT |
| UNC5B | CAGCCTAGATGCCCCCAACTCA | TCCCAGAGGTCCAGGATCACAC |
| VEGFR1 | CCACCTCCATGTTTGATGACTACC | CAGCCCCGACTCCTTACTTTTACTG |
| VEGFR2 | CCAGATGACAACCAGACGGACAG | GGCACCATTCCACCAAAAGATG |
| VEGFR3 | GCCAGGTATTACAACTGGGTGTCCT | TCTGGTTGTCCACAGAGCCTTTGT |

| Gene (chicken) | Fw | Rv |
| --- | --- | --- |
| PDGFRA | ATCTCAGCGTTGTTTGCCA | AATTCTATCCATCAAAGTCATAGG |
| PDGFRB | AAGGATAGGCGATGTCATGG | ATACCTGTCAGAGTGAGTGGGG |

**References**

1. Crampton, S. P., Davis, J. & Hughes, C. C. W. Isolation of human umbilical vein endothelial cells (HUVEC). *J. Vis. Exp.* **3,** 183 (2007).

2. Tigges, U., Welser-Alves, J. V., Boroujerdi, A. & Milner, R. A novel and simple method for culturing pericytes from mouse brain. *Microvasc. Res.* **84,** 74–80 (2012).

3. Thijssen, V. L. J. L., Brandwijk, R. J. M. G. E., Dings, R. P. M. & Griffioen, A. W. Angiogenesis gene expression profiling in xenograft models to study cellular interactions. *Exp. Cell Res.* **299,** 286–293 (2004).

4. Ruijter, J. M., Ruiz Villalba, A., Hellemans, J., Untergasser, A. & van den Hoff, M. J. B. Removal of between-run variation in a multi-plate qPCR experiment. *Biomol. Detect. Quantif.* **5,** 10–14 (2015).
